# Efficacy optimization of low frequency microbubble-mediated sonoporation as a drug delivery platform to cancer cells

**DOI:** 10.1016/j.ijpx.2022.100132

**Published:** 2022-09-22

**Authors:** Michal Eck, Ramona Aronovich, Tali Ilovitsh

**Affiliations:** Department of Biomedical Engineering, Tel Aviv University, Tel Aviv 6997801, Israel

**Keywords:** FUS, Low frequency, Microbubbles, Sonoporation, Drug delivery

## Abstract

Ultrasound insonation of microbubbles can be used to form pores in cell membranes and facilitate the local trans-membrane transport of drugs and genes. An important factor in efficient delivery is the size of the delivered target compared to the generated membrane pores. Large molecule delivery remains a challenge, and can affect the resulting therapeutic outcomes. To facilitate large molecule delivery, large pores need to be formed. While ultrasound typically uses megahertz frequencies, it was recently shown that when microbubbles are excited at a frequency of 250 kHz (an order of magnitude below the resonance frequency of these agents), their oscillations are significantly enhanced as compared to the megahertz range. Here, to promote the delivery of large molecules, we suggest using this low frequency and inducing large pore formation through the high-amplitude oscillations of microbubbles. We assessed the impact of low frequency microbubble-mediated sonoporation on breast cancer cell uptake by optimizing the delivery of 4 fluorescent molecules ranging from 1.2 to 70 kDa in size. The optimal ultrasound peak negative pressure was found to be 500 kPa. Increasing the pressure did not enhance the fraction of fluorescent cells, and in fact reduced cell viability. For the smaller molecule sizes, 1.2 kDa and 4 kDa, the groups treated with an ultrasound pressure of 500 kPa and MB resulted in a fraction of 58% and 29% of fluorescent cells respectively, whereas delivery of 20 kDa and 70 kDa molecules yielded 10% and 5%, respectively. These findings suggest that low-frequency (e.g., 250 kHz) insonation of microbubbles results in high amplitude oscillation in vitro that increase the uptake of large molecules. Successful ultrasound-mediated molecule delivery requires the careful selection of insonation parameters to maximize the therapeutic effect by increasing cell uptake.

## 1 Introduction

In sonoporation, ultrasound (US) waves generate cavitation to induce pores in cell membranes. Most sonoporation methods utilize microbubble (MB) contrast agents. MBs are spherical and are composed of a gas core and a stabilizing shell. Upon US application, the MBs act as cavitation nuclei that enhance cavitation-induced bioeffects, including membrane permeabilization (Yang et al., 2020; Couture et al., 2014). This process forms transient pores in cell membranes that enable the delivery of non-permeable extracellular molecules including drugs, proteins, genes and a variety of therapeutic agents (Singh et al., 2016). The use of cell-targeted MB (TMB) further enhances the sonoporation effect, since the close proximity between the adherent MB and the cell surface improves the efficacy of pore creation (Lentacker et al., 2014; Kooiman et al., 2011). Upon US excitation, MBs oscillate, thus increasing cell membrane permeability and providing a conduit for the delivery of different therapeutics into the target region. Stable cavitation is said to exist when the MB oscillations are relatively small around an equilibrium volume. When a certain pressure threshold has been reached, inertial cavitation sets in. During inertial cavitation, there is significant MB expansion, leading to MB collapse and fragmentation, which can result in a more aggressive biophysical effect (Ilovitsh et al., 2018; Fan et al., 2014).

Sonoporation efficacy depends on the cell type, the size of the generated pore, and the delivered particle size, among other parameters. The pore size is affected by factors such as the MB concentration, formulation, targeting method, treatment duration, and US parameters (Langeveld et al., 2022; Wawryka et al., 2019). These parameters also affect the duration of the opened pore and the end result of successful delivery or cell death (Ferrara et al., 2007; Van Wamel et al., 2006). Enlarging the pores by enhanced MB oscillations is likely to increase cellular uptake via sonoporation.

**Fig. 1:**
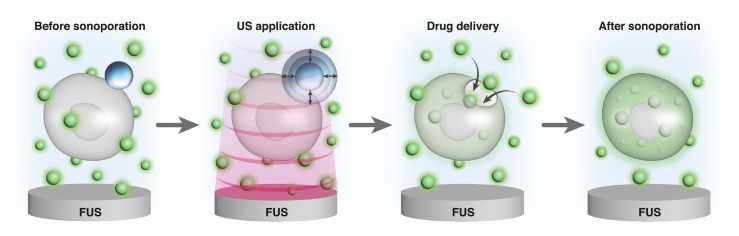
Targeted microbubble-mediated sonoporation of cancer cells illustration

Earlier works assumed that MB oscillations are enhanced at their resonance frequency (Stride, 2009). The resonance frequency depends on factors such as the MB diameter, composition and surrounding medium (Chowdhury et al., 2020), and is typically above 1 MHz (Qin and Ferrara, 2007). For these reasons, MB-mediated sonoporation optimization studies applied insonation at frequencies of 500 kHz and above, although most were above 1 MHz (Karshafian, 2010; Duvshani-Eshet and Machluf, 2005; Li et al., 2009; Meijering et al., 2007; Telichko et al., 2021). However, more recent studies have shown that at a low frequency of 250 kHz, which is an order of magnitude below the MB resonance frequency, enhanced MB oscillations are observed and can increase sonoporation efficacy (Ilovitsh et al., 2018; Ilovitsh et al., 2020). The physical mechanism accounting for this phenomenon is known as the Blake threshold effect, which states that when exciting a MB well below its resonance frequency, beyond a defined threshold pressure (Blake threshold), the MB will expand extensively (Harkin et al., 1999; Blake, 1949; Lauterborn, 1976). These high amplitude oscillations can transform the TMB into mechanical therapeutic warheads that poke large holes in cell membranes (Fig. 1. This effect was recently shown to facilitate the delivery of large genes into cancer cells (Ilovitsh et al., 2020), and for low energy mechanical ablation of tumors (Bismuth et al., 2021; Glickstein et al., 2022). Given the advantages of low frequency insonation, and the growing interest in the use of low frequencies (Ilovitsh et al., 2020; Bai et al., 2016; Zhang et al., 2021), there is a crucial need for studies on ways to optimize the relationship between molecule size and delivery efficacy upon low frequency sonoporation for effective drug delivery. This study assessed the impact of particle size on cell uptake by directly comparing the delivery of 4 molecules with different molecular weights. Most previous sonoporation studies have examined the delivery of small molecules such as Propidium Iodide (PI) (Fan et al., 2012; Meng et al., 2019; Wang et al., 2018; Beekers et al., 2020; Helfield et al., 2016; Park et al., 2011; Skachkov et al., 2014); however, the delivery of small molecules does not fully represent the ability to deliver larger particles. Hence, this study examined the sonoporation effect on molecules ranging from 1.2 kDa up to 70 kDa. This size range corresponds to materials such as chemotherapeutic drugs and drug carriers (1–70 kDa), siRNA (∼14 kDa) and proteins (3–40 kDa) (Maciulevičius et al., 2022; Liu et al., 2019; Dreher et al., 2006; Matsunaga et al., 2018; Wang et al., 2016; Tsai et al., 2012; Tian et al., 2020; Delalande et al., 2013; Huang et al., 2017; Marmottant et al., 2005).

Further, the use of a low frequency reduces the pressure needed to achieve effective delivery. As a result, the spatiotemporal precision of the method is improved, since only the MBs and the target tissues are affected, while the healthy surrounding tissue remains unaffected. Moreover, the low energies do not induce heat, so that the effect remains purely mechanical. The additional benefits of the use of low frequency have to do with its large penetration depth as a result of low attenuation compared to high frequencies, which is likely to facilitate the treatment of deep-seated tissues and organs.

The main focus here centers on breast cancer cell sonoporation. Their sonoporation is often more challenging than other cell types, such as muscle cells, where small MB oscillations suffice for robust sonoporation (Tsai et al., 2012). In addition, drug delivery to cancer cells has a high clinical importance. Currently, cancer treatment involves invasive surgery combined with adjuvant therapy such as chemo- or immuno-therapy. However, the lack of specificity of these adjuvant therapies and their toxic nature triggers systemic toxic side effects in healthy tissues (Tian et al., 2020). By contrast, TMB-mediated sonoporation is a site specific targeted delivery method (Fan et al., 2014; Delalande et al., 2013). Moreover, reducing tumor burden plays a key role in the success of adjuvant therapies (Huang et al., 2017). Along with enhancing drug delivery, low frequency insonation of MBs can reduce cancer cell viability, as a combined strategy for cancer treatment.

## 2 Materials and methods

### 2.1 Numerical modeling

The MB expansion ratio was estimated using the Marmottant model (Marmottant et al., 2005) which exhibits good fit with experimental observations (Ilovitsh et al., 2018; Sojahrood et al., 2012). The simulation was implemented in Matlab (version 2017b, The MathWorks, Natick, MA, USA), and took the MB composition, the viscosity and density of the surrounding medium, and the US excitation parameters into account. The MB expansion ratio, defined as the maximal diameter divided by the resting MB diameter, was evaluated for a center frequency of 250 kHz and peak negative pressures (PNP) ranging from 0 to 800 kPa. The simulation parameters were described previously (Glickstein et al., 2022). The initial MB resting diameter was set to 1.5 μm.

### 2.2 Microbubble preparation

TMBs composed of a phospholipid shell and a perfluorobutane (C_4_F_10_) gas core were used in this study and prepared as reported previously (Ilovitsh et al., 2020). Briefly, for the TMB preparation, the following lipids (2.5 mg per 1 ml) were combined in a 90:5:5 M ratio: distearoylphosphatidylcholine (DSPC), 1,2-distearoyl-sn-glycero-3-phosphoethanolamine-N- [methoxy(polyethylene glycol)-2000] (ammonium salt) (DSPE-PEG2K), and 1,2-distearoylsnglycero- 3-phosphoethanolamine-N-[biotinyl(polyethylene glycol)2000] (DSPE-PEG2000-Biotin). All lipids were purchased from Sigma-Aldrich. The lipids were sonicated at 62 °C with a 7.4 pH buffer solution (80% NaCl, 10% glycerol, and 10% propylene glycol) and aliquoted into 2 ml vials (1 ml liquid in each). After sealing the vial, the air within the 1 ml gap in the vial was replaced by perfluorobutane gas. This was done by inserting a perfluorobutane gas syringe with a 21G needle into the vial, and slowly purging it inside. In parallel, 27G needle was inserted through the rubber cap, into the top of the vial. This needle was used as an outlet for the excess air and perfluorobutane gas. The vials were kept at 4 °C until use. Upon use, to activate the TMB, the vial was shaken for 45 s in a VIALMIX shaker. Then, using centrifugation, the TMB were purified by removing most of the TMBs with radii smaller than 0.5 μm. 400 μg of streptavidin (Thermo Fisher Scientific, S888) was added to the TMB which were then incubated on a rotator for 25 min. After the 25- min incubation period the TMB were purified again using centrifugation to remove excess streptavidin. Then, 15 μg of Biotin anti-mouse CD326 antibody (EpCAM, BLG-118204) was added to the TMB, which were incubated again on a rotator for 25 min and then purified in the same way to remove the excess antibody. The TMB size and concentration were measured using a particle counter system (AccuSizer FX-Nano, Particle Sizing Systems, Entegris, MA, USA).

The amount of streptavidin and CD326 antibody per TMB was estimated as follows: the DSPC headgroup has an average area of 0.6 nm^2^ (Huang and Mason, 1978; Kučerka et al., 2004; Ilovitsh et al., 2020); thus, a MB with an average diameter of 1.5 μm has ∼10^7^ lipid molecules/shell [π*(1.5×10^−6^)^2^/(0.6×10^−18^) ≅ 10^7^]. 5% of the total lipids are DSPE-PEG-Biotin; therefore, each MB has 5*10^5^ DSPE-PEG2K-Biotin. We assume that one biotin-lipid conjugates with one streptavidin and therefore each MB has ∼5×10^5^ streptavidin. On average, assuming that two antibody molecules bind to one streptavidin, each TMB has ∼10×10^5^ antibody molecules (Shi et al., 2013).

### 2.3 Cell preparation

4T1 cells, murine tumor-derived cell line (purchased from ATCC), were used in all experiments. The cells were cultured in T75 tissue culture flasks containing RPMI 1640 L-Glutamine (+) with 10% fetal bovine serum (FBS) and 1% penicillin−streptomycin (P/S) at 37 °C in a humidified 5% CO2 incubator. Upon use, when cell confluency reached 85%, the cells were dissociated using TrypLE Express (Thermo Fisher Scientific, 12604021) and resuspended at a concentration of 2 × 10^6^ cells in 300 μl degassed PBS (calcium(+), magnesium(+)).

### 2.4 Ultrasound setup

**Fig. 2:**
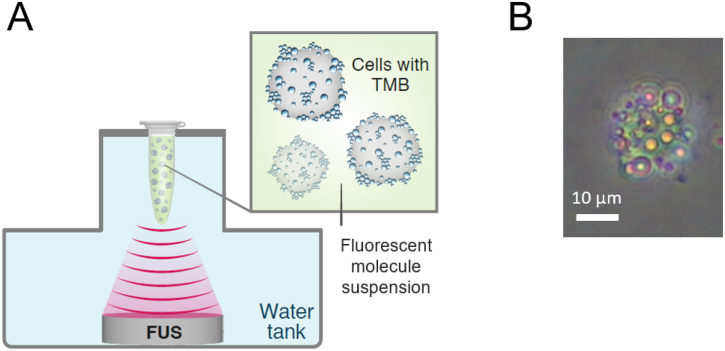
Microbubble-mediated sonoporation setup. (A) An ultrasound transducer is placed at the bottom of a water tank and a tube containing cells with TMB in a fluorescent molecule suspension is placed at the transducer's focal spot. (B) A microscope image showing a cell with TMBs attached to the cell, at 20× magnification.

For the US treatment, a 0.5 ml Eppendorf tube with cell suspension was positioned at the focal spot of a spherically focused single-element transducer (H115, Sonic Concepts, Bothell, WA, USA), which was placed facing upward at the bottom of a degassed water tank (Fig. 2A). The transducer was focused to a distance of 45 mm. Each tube was insonated at a center frequency of 250 kHz and PNP that ranged from 100 to 800 kPa for a duration of 30 s.

### 2.5 Sonoporation experiments

Fluorescent molecules ranging from 1.2 kDa to 70 kDa were used to investigate delivery to the cells. Each experiment included one of the following materials: 7-aminoactinomycin D (7-AAD), a 1.27 kDa fluorescent dye that undergoes a spectral shift upon association with DNA (Thermo Fisher Scientific, A1310), Fluorescein isothiocyanate–dextran average molecular weights 4 kDa (FITC-Dextran 4) (46944, Sigma-Aldrich), FITC-Dextran 20 kDa (Sigma-Aldrich, FD20), and FITC-Dextran 70 kDa (Sigma-Aldrich, 46945). After cell preparation, TMB were added to the cell mixture at a ratio of 50 TMB per cell (Bismuth et al., 2021) and incubated for 20 min at room temperature on a rotator allowing the TMB to bind to the cells. Following incubation, microscopy imaging was used to confirm the TMB binding to the cells (Fig. 2B, Fig. S1). For the same targeted microbubbles concentration used here, we previously found that 19.4 ± 3 TMBs were bound per cell. This is a 38.8 ± 6% effective binding rate (Bismuth et al., 2021).

For the 7-AAD sonoporation experiments the incubation was performed separately for each group, prior to which the cells were kept on ice. For the FITC sonoporation experiments, the incubation was performed for all groups at the same time.

Following incubation, the mixture of cells and TMB was aliquoted into 0.5 ml Eppendorf tubes, along with degassed PBS +/+ and the sonoporated material (5 μg/ml 7-AAD or 1 mg/ml FITC-dextran). For experiments with 7-AAD and FITC-Dextran 4, due to the molecules' small size, the US treatment was performed immediately after adding the material to the 0.5 ml Eppendorf tube. For the FITC-Dextran 20 and 70, the cell mixture was incubated for 30 min with the materials prior to US treatment.

For the 7-AAD sonoporation experiments, following US treatment, 10 μg/ml of Hoechst (33342, Abcam) was added to the tube to quantify the total number of cells in the sample. The suspension was transferred from the Eppendorf tube to a 35 mm cell culture dish (430165, Corning) and viewed under fluorescence microscope using BF, an mCherry filter and a DAPI filter at 10× magnification. Seven frames from different locations of the culture dish were saved to be analyzed further.

For the FITC-Dextran sonoporation experiments, following US treatment, the suspension was transferred from the Eppendorf tube to a 24-well plate (3526, Corning) which was pre-prepared with 300 μl of culture media with an additional 1.5% P/S and then incubated for 24 h at 37 °C in a humidified 5% CO2 incubator.

After incubation for 24 h, each well was washed 3 times with PBS to remove the remaining FITC suspension and then media were added to each well. Imaging and quantification of the fraction of fluorescent cells was performed using IncuCyte Live-Cell Analysis System (Essen Bioscience). All experiments were performed in triplicate for each group.

### 2.6 Viability experiments

Viability was calculated as the percentage of remaining cells in each group compared to the sham group. For the 7-AAD experiments, viability was measured immediately after US treatment using CellDrop device (DeNovix). For the FITC experiments, viability was measured at 24 h and at 72 h post-US treatment. These time points were selected in order to match the time points of the molecules uptake experiments. An additional viability test was performed 72 h post treatment to evaluate cells recovery as a function of time.

Following US treatment, the suspension was transferred from the Eppendorf tube to a 24-well plate (Corning, 3526) or 6-well plate (Corning, 3516), which were pre-prepared with either 300ul or 2 ml of culture media with an additional 1.5% P/S and then incubated for 24 h at 37 °C in a humidified 5% CO2 incubator. After 24 h of incubation, each well was washed with PBS, then dissociated with TrypLE and counted using the CellDrop device (DeNovix). For the three day viability test, the FITC suspension was washed after one day and then the cells were incubated with media for the remaining time until count.

### 2.7 Analysis

For the 7-AAD sonoporation experiments, microscope images were analyzed using ImageJ software. Each image was uploaded to the ImageJ software, the image type was changed to 16-bit, the threshold was adjusted to view the stained cells clearly and the background was removed. Each experiment was conducted in triplicate, and 7 images were acquired in each repetition, for a total of 21 images for each group.

The number of Hoechst stained cells (Blue) was defined as the total number of cells in the sample. The number of red cells was the number of cells stained with 7-AAD. The fraction of fluorescent cells was calculated as the percentage of 7-AAD stained cells divided by the number of Hoechst stained cells.

For the FITC sonoporation experiments, the analysis was performed using IncuCyte Live-Cell Analysis System (Essen Bioscience). Each well was sampled 25 times at 20× magnification. The calculation used the green confluence (green cell area) normalized by the phase confluence (total cell area). This required configuring the analysis to distinguish cells and the same fluorescence threshold for all images.

GraphPad Prism 9 software was used for the statistical analysis. *P* values <0.05 were considered significant and were adjusted for multiple comparisons as indicated in the figure captions. The results are presented as the mean ± SD.

## 3 Results

### 3.1 Microbubble numerical simulations

**Fig. 3:**
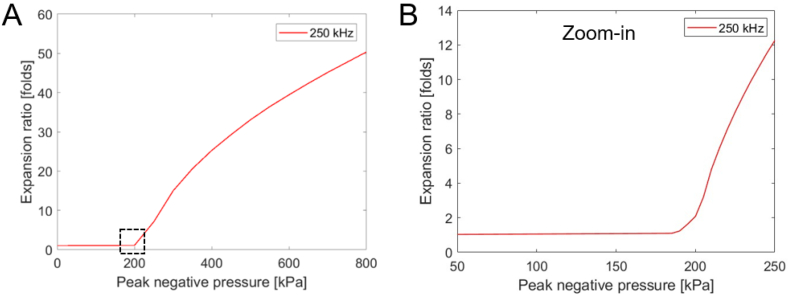
Predicted MB expansion ratio. Microbubble expansion ratio as a function of the peak negative pressure for 250 kHz and a MB initial diameter of 1.5 μm, for a peak negative range of: (A) 0–800 kPa, and (B) Zoom-in on peak negative pressures of up to 250 kPa.

The MB expansion ratio provided a metric to assess its vibrational response. Expansion ratios from 1.1 to 3.5 were considered to reflect stable cavitation, while inertial cavitation was defined as initiating at expansion ratios of >3.5 fold (Ilovitsh et al., 2018). The size of the generated pore in the cell membrane also depends on the MB expansion ratio. Therefore, to determine the range of PNP for the operation, numerical simulations of the MB expansion ratio were conducted for a center frequency of 250 kHz as a function of the PNP (0 to 800 kPa) (Fig. 3A). MB oscillations are enhanced at a low frequency of 250 kHz, and therefore the stable cavitation range is narrow (Ilovitsh et al., 2018). Based on these numerical simulations, the predicted stable cavitation range occurs between 85 and 205 kPa (Fig. 3B). Above this PNP, a steep increase in the expansion ratio initiated and reached factors of 15, 33, and 50 for a PNP of 300, 500 and 800 kPa, respectively.

### 3.2 Sonoporation results

The delivery of four different sized impermeable dye molecules following low frequency US insonation at a center frequency of 250 kHz was optimized, and directly compared.

### 3.3 Sonoporation of 7-AAD

**Fig. 4:**
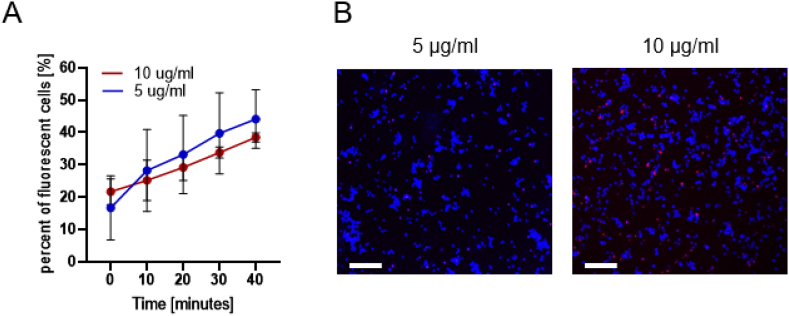
7-AAD sham group over time. (A) 7-AAD fluorescent cells as percentage out of total live cells in the sham group over time. (B) Fluorescence microscopy images for 7-AAD fluorescent cells without any treatment (sham) for concentration of 5 μg/ml (left) and 10 μg/ml (right). Hoechst stained cells (total cells in the sample) marked in blue and 7-AAD positive cells appear as pink. Scale bar is common to all subfigures in (B) and is 200 μm. (For interpretation of the references to colour in this figure legend, the reader is referred to the web version of this article.)

The first molecule tested was the 7-AAD, which has a molecular weight of 1.2 kDa. Based on a previous study (Zembruski et al., 2012), two 7-AAD concentrations were tested (5 μg/ml and 10 μg/ml), to assess the percentage of fluorescent cells without US and MB treatment (sham). The fraction of fluorescent cells in the sham group, as observed via fluorescence microscopy, increased as a function of incubation time (Fig. 4A). Therefore, in the following experiments, a time point of 0 (e.g. immediately post-US treatment) was used. For this time point, the fraction of fluorescent cells for both concentrations was similar: 16.8 ± 7.7% for 5 μg/ml and 21.6 ± 3.2% for 10 μg/ml (Fig. 4B). Therefore, a concentration of 5 μg/ml was chosen for the subsequent US and TMB sonoporation experiments.

**Fig. 5:**
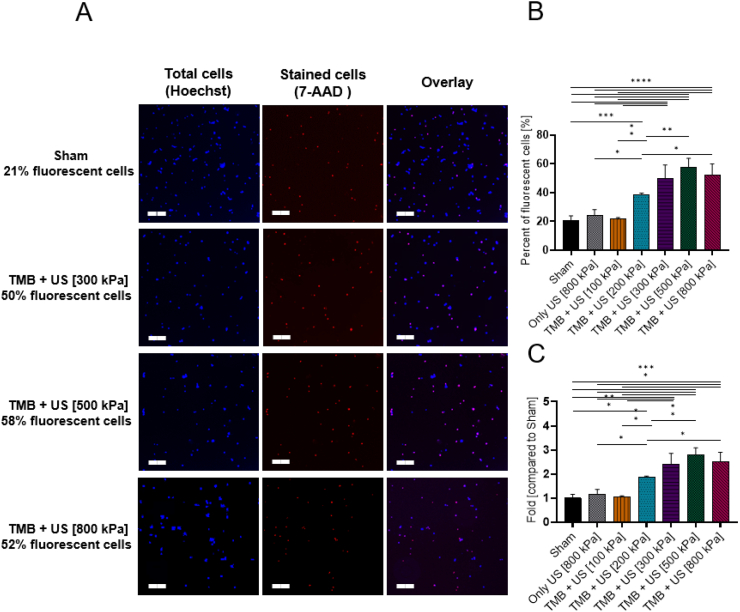
7-AAD sonoporation (A) Fluorescence microscopy images for different ultrasound treatment groups. Each row presents a different PNP immediately following sonoporation. Left column is the Hoechst stained cells, middle column is the 7-AAD stained cells, and right column is an overlay of both Hoechst and 7-AAD stained cells. Images were acquired with 10× magnification. Scale bar is common to all subfigures in (A) and was 200 μm. (B) 7-AAD stained cells expressed as the percentage of live cells for the different treatment and control groups. (C) The graph in (B) presented as the fold uptake compared to the sham group. (B and C) One-way ANOVA with Tukey's multiple comparison test. Adjusted *p* values were *p < 0.05, **p < 0.01, ***p < 0.001, ****p < 0.0001. All data are plotted as the mean ± SD.

The fraction of 7-AAD stained cells increased as a function of the applied PNP. The percent of fluorescent cells was similar between the sham (21 ± 3%), US only (24 ± 3.3%) and treatment with TMB and US at 100 kPa (21.5 ± 0.9%). The fraction of fluorescent cells reached a maximal value of 57.7 ± 4.9% for 500 kPa (Fig. 5B). Increasing the PNP to 800 kPa reduced the fraction of fluorescent cells to 52 ± 6.3% (non-significant, *p* > 0.05). This suggests that 500 kPa was the optimal delivery pressure for this molecule size. The results, presented as folds, increased compared to the sham group and showed a maximal value of 2.8 ± 0.23 fold compared to the sham group (Fig. 5C).

### 3.4 Sonoporation of FITC 4 kDa

**Fig. 6:**
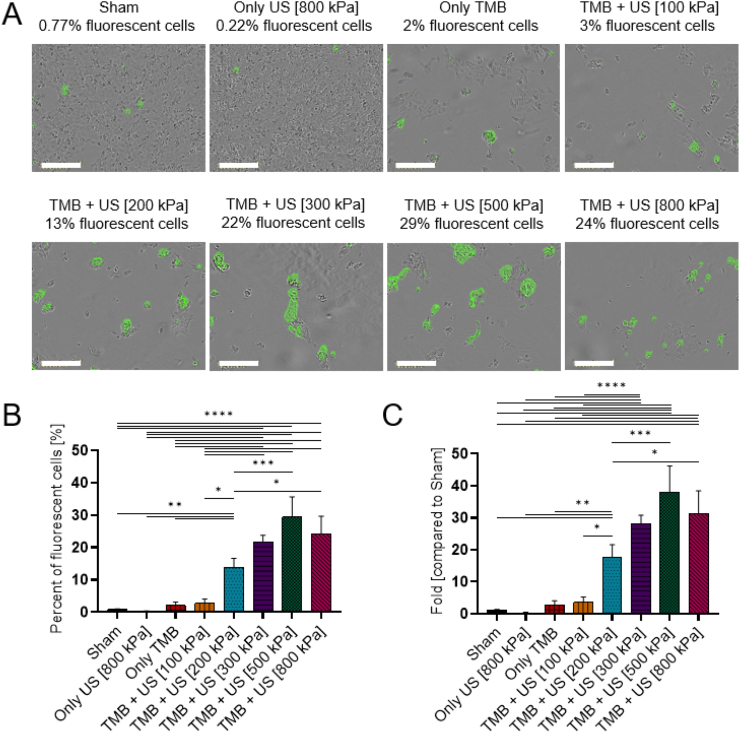
FITC 4 kDa sonoporation. (A) Overlay images of cells and FITC 4 kDa for different ultrasound treatment groups. Each image presents a different PNP 1 day after sonoporation. Images were acquired by the Incucyte system with 20× magnification. Scale bar is common to all subfigures in (A) and is 200 μm. (B) FITC 4 kDa stained cells expressed as the percentage of live cells for the different treatment and control groups. (C) The graph in (B) presented as fold uptake compared to the sham group. (B and C) One-way ANOVA with Tukey's multiple comparison test. Adjusted *p* values were *p < 0.05, **p < 0.01, ***p < 0.001, ****p < 0.0001. All data are plotted as the mean ± SD.

The next molecule that was tested was 4 kDa FITC-dextran. The same FITC-dextran concentration of 1 mg/ml was used for all FITC-dextran sizes (4, 20 and 70 kDa) and was chosen based on previous studies (Miller et al., 1999; Karshafian et al., 2010; Karshafian et al., 2009; Meijering et al., 2009). FITC 4 kDa exhibited the same delivery trend as 7-AAD, where the fraction of fluorescent cells increased as a function of the applied PNP, up to a PNP of 500 kPa (maximal fraction of fluorescent cells was 29.39 ± 5.07%). Beyond this PNP, the fraction of fluorescent cells began to reduce to 24.14 ± 4.46% for a PNP of 800 kPa (not significant, *p* > 0.05) (Fig. 6). A similar percentage was observed in the control groups of sham (0.77 ± 0.21%) and only US (0.22 ± 0.12%). In addition, the control group of only TMB (2.05 ± 0.86%) had a similar percent of fluorescent cells as the treated group with 100 kPa. These results suggest that 500 kPa was the optimal PNP for the delivery of these molecules, consistent with the 7-AAD results. Specifically, in terms of the fold increase compared to the sham group, the maximal uptake for 500 kPa represented a 38 ± 6.5 fold increase compared to sham group, which was significantly higher than the factor obtained for the 7-AAD molecule (Fig. 6C).

### 3.5 Sonoporation of FITC 20 kDa

**Fig. 7:**
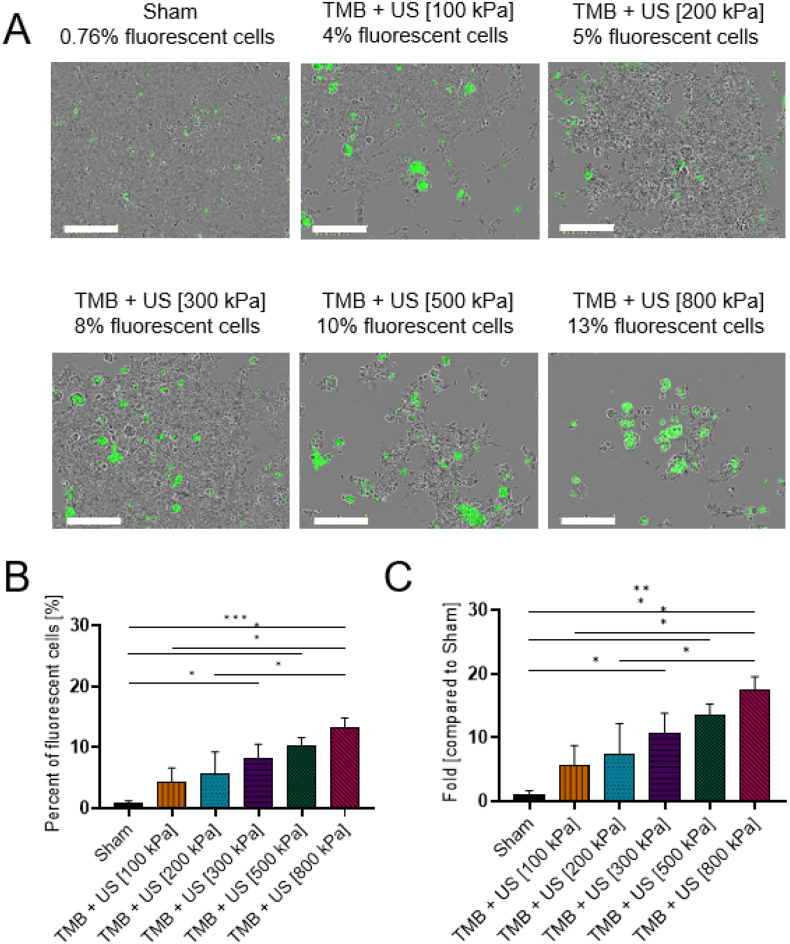
FITC 20 kDa sonoporation. (A) Overlay images of cells and FITC 20 kDa for different ultrasound treatment groups. Each image presents a different PNP 1 day after sonoporation. Images were acquired by the Incucyte system with 20× magnification. Scale bar is common to all subfigures in (A) and is 200 μm. (B) FITC 20 kDa stained cells expressed as the percentage of live cells for the different treatment and control groups. (C) The graph in (B) presented as fold uptake compared to the sham group. (B and C) One-way ANOVA with Tukey's multiple comparison test. Adjusted *p* values were *p < 0.05, **p < 0.01, ***p < 0.001, ****p < 0.0001. All data are plotted as the mean ± SD.

Next, the size of the delivered molecule was increased to FITC-dextran 20 kDa (Fig. 7). The percent of fluorescent cells increased as a function of the applied PNP. Maximal percentage was observed for a PNP of 800 kPa (13.23 ± 1.32%), while for the 500 kPa the fraction of fluorescent cells was 10.34 ± 1.03% (non- significant, *p* > 0.05). A fraction of fluorescent cells of 0.76 ± 0.41% was seen for the sham group and increased for the 100 kPa (4.34 ± 1.86%), 200 kPa (5.57 ± 3.01%), and 300 kPa (8.14 ± 1.95%) (Fig. 7B). The maximal fold increase for the 800 kPa PNP compared to the sham group was 17.4 ± 1.74 (Fig. 7C).

### 3.6 Sonoporation of FITC 70 kDa

**Fig. 8:**
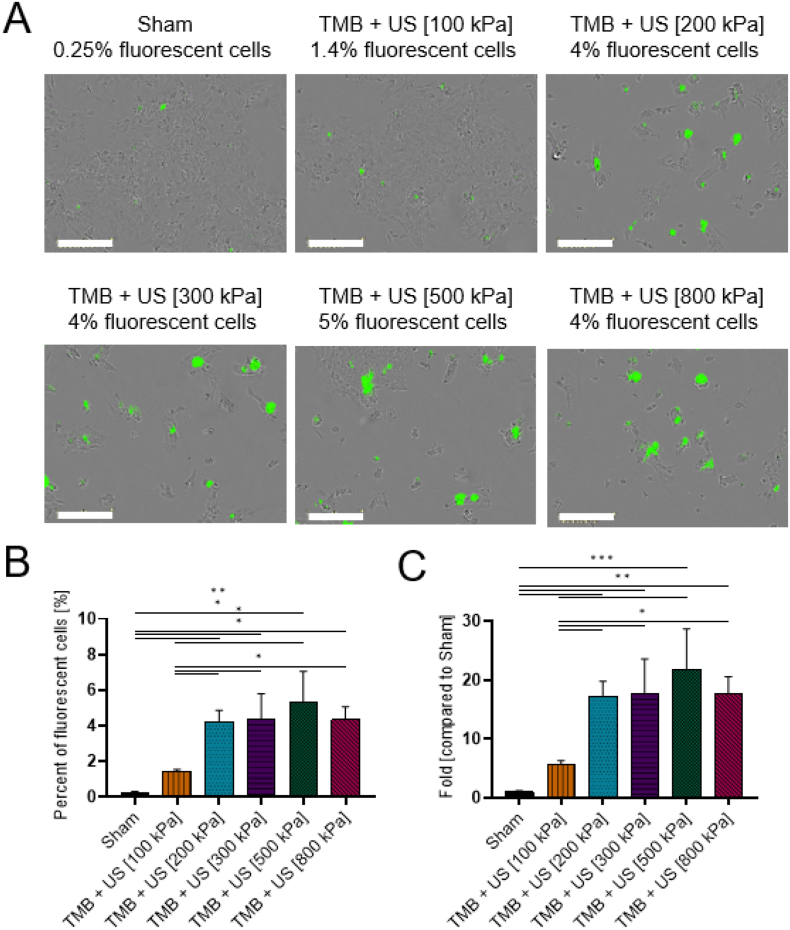
FITC 70 kDa sonoporation. (A) Overlay images of cells and FITC 70 kDa for different ultrasound treatment groups. Each image presents a different PNP 1 day after sonoporation. Images were acquired by the Incucyte system with 20× magnification. Scale bar is common to all subfigures in (A) and is 200 μm. (B) FITC 70 kDa stained cells expressed as the percentage of live cells for the different treatment and control groups. (C) The graph in (B) presented as fold uptake compared to the sham group. (B and C) One-way ANOVA with Tukey's multiple comparison test. Adjusted *p* values were *p < 0.05, **p < 0.01, ***p < 0.001, ****p < 0.0001. All data are plotted as the mean ± SD.

The largest molecule that was tested was FITC-dextran 70 kDa (Fig. 8). For this molecule size, 0.24 ± 0.03% fluorescent cells was observed in the sham group and 1.4 ± 0.12% for treatment with 100 kPa. A similar percentage was obtained for treatment with 200 kPa (4.22 ± 0.52%) and 300 kPa (4.33 ± 1.19%). Maximal percent of fluorescent cells was observed for 500 kPa (5.3 ± 1.4%) and dropped to 4.32 ± 0.6% for 800 kPa (not significant, *p* > 0.05), consistent with the trends observed for the delivery of 7-AAD and FITC 4 kDa. The maximal fold increase in uptake for treatment with 500 kPa was 21.5 ± 5.7 compared to the sham group.

### 3.7 Viability experiments

**Fig. 9:**
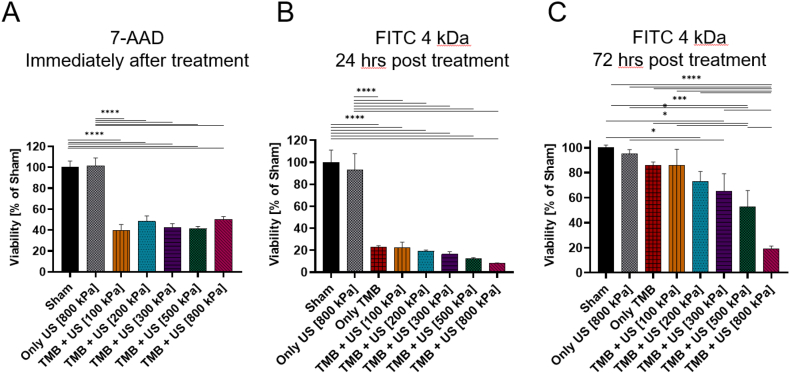
Cell viability post treatment. (A) Viability of cells expressed as the percentage of the sham group for the different treatment and control groups immediately after sonoporation with 7-AAD. (B) Viability of cells expressed as the percentage of sham group for the different treatment and control groups 1 day after sonoporation with FITC 4 kDa. (C) Viability of cells expressed as the percentage of sham group for the different treatment and control groups 3 days after sonoporation with FITC 4 kDa. One-way ANOVA with Tukey's multiple comparison test. Adjusted *p* values were *p < 0.05, **p < 0.01, ***p < 0.001, ****p < 0.0001. All data are plotted as the mean ± SD.

Cell viability was evaluated for the treatment groups at multiple time points following sonoporation. For the 7-AAD molecule, cell viability was assessed immediately after sonoporation and demonstrated a similar viability of ∼40% live cells for the different treatment groups. The Only US group had the same results as the sham group (Fig. 9A).

Unlike the 7-AAD, where the molecule uptake was assessed immediately after sonoporation, the FITC molecule uptake was evaluated 24 h post sonoporation, Therefore, cell viability was assessed 24 h post- sonoporation as well. For treatment with FITC 4 kDa, no significant difference was observed between the sham (100 ± 9.04%) and Only US (93.19 ± 11.9%) groups. In the treatment groups, viability dropped to 22.87 ± 1.01% for treatment only with TMB (no US application). Application of US + TMB further reduced viability from 22.57 ± 3.9% at a PNP of 100 kPa to 12.1 ± 0.92% and 7.92 ± 0.42% for treatment with 500 and 800 kPa, respectively (Fig. 9B). Cell recovery was assessed by evaluating cell viability 72 h. post sonoporation. Viability was significantly increased at 72 h compared to 24 h for all treated groups, reaching 52.84 ± 10.5% for the 500 kPa treatment. The sham and Only US groups remained similar, as did the Only TMB group (85.83 ± 2.17%) and the TMB with 100 kPa US (85.73 ± 1.67%). (Fig. 9C).

## 4 Discussion

Sonoporation is a growing field which has documented advantages over other delivery methods such as electroporation and viral vectors (Juffermans et al., 2001). The clinical applications of sonoporation include drug and gene delivery for different cell types and locations (Ilovitsh et al., 2020; Dreher et al., 2006; Wang et al., 2016; Sitta and Howard, 2021; Lin et al., 2010; Karki et al., 2019; Xu et al., 2014). To enhance this therapeutic platform, a better understanding of the relationship between the insonation parameters and the delivered material size is crucial. For example, sonoporation can be used to increase the spatiotemporal precision of the delivery of anti-cancer drugs. To do so, a large number of cancer cells need to be sonoporated to successfully impact the majority of the cancer cells, and a high delivery efficacy is required to maximize the drug concentration within the cells. Most optimization studies in this field have implemented frequencies exceeding 1 MHz. Recent studies show the advantages of using lower frequencies. The use of a center frequency of 250 kHz is particularly important, since these systems have entered clinical use. This low frequency advantages lie in the large focal spot that enables to treat a large volume of the tumor simultaneously, the increased penetration depth, its reduced attenuation and distortion, and improved beam steering capabilities. Notably, for brain therapy applications, the lower frequency aids in focusing through the human skull with minimal distortion and attenuation. Low frequency US was used to treat over 500 patients for brain indications applications. Our lab uniquely uses the high amplitude MB oscillations that occur at this low frequency to develop mechanically-induced bioeffects at low PNPs, such as low energy bubble-mediated histotripsy (Bismuth et al., 2021; Bismuth et al., 2022). Here, we showed that coupled with low frequency excitation, MBs can be used as mechanical therapeutic warheads that generate sufficiently large pores in cancer cell membranes to deliver large molecules. To the best of our knowledge, most of the sonoporation studies are performed at megahertz frequencies where strong MB oscillations require PNP that exceed the FDA safety threshold.

Higher frequencies studies have focused on the delivery of a single molecule with a constant diameter. Here we assessed the impact of molecule weight on delivery efficacy at a low frequency of 250 kHz, while using the same setup, to facilitate a direct comparison between molecules. Four molecules with molecular weights of 1.2, 4, 20 and 70 kDa were tested. These molecular weights are clinically important. The smaller molecular weights represent small anticancer drugs such as chemotherapy (<1 kDa) (Maciulevičius et al., 2022; Jackson et al., 2011). 20 kDa molecules, for example, match siRNA and miRNA (∼14 kDa) (Karki et al., 2019; Ha et al., 2015; Kinoshita and Hynynen, 2005; Mullick Chowdhury et al., 2016), whereas 70 kDa corresponds to the size of proteins, macromolecules and genes (Liu et al., 2019; Dreher et al., 2006; Matsunaga et al., 2018; Wang et al., 2016). Since the hydrodynamic radius of the molecule is not directly proportional to its molecular mass, the delivery of other types of molecules with a similar molecular weight but with a different shape, might require further optimization. In the case of cancer therapy, alongside high delivery efficacy, the associated goal is to reduce cell viability, since minimizing the tumor burden plays an important role in the success of cancer treatment (Huang et al., 2017). The high MB expansion ratio caused by low frequency insonation makes it possible to reduce cell viability while creating large pores in cell membranes that facilitate drug delivery. Although this article dealt with the sonoporation of cancer cells, future applications could include the sonoporation of other cell types, such as immune cells, muscle cells and endothelial cells (Karki et al., 2019).

Numerical simulations were performed to estimate the required PNPs. The results indicated that above a PNP of 200 kPa, inertial cavitation occurs, which is likely to increase cellular uptake as a result of pore formation via TMB collapse. The experimental results confirmed the theoretical predictions. For all molecule sizes, a large increase in the fraction of fluorescent cells was observed at a PNP of 200 kPa, compared to 100 kPa where the MBs were predicted to oscillate in stable cavitation. This clearly suggests that inertial cavitation plays a significant role in the successful sonoporation of cancer cells, unlike other cell types such as muscle cells where stable cavitation suffices (Tsai et al., 2012).

Fluorescent cells percentage was the highest for the smallest molecules, with ∼58% of live cells for the 7-AAD molecule with a molecular weight of 1.2 kDa, at a PNP of 500 kPa. For the same PNP, the fraction of fluorescent cells was 29, 10 and 5% of live cells for FITC molecules with a molecular weight of 4, 20 and 70 kDa, respectively. However, when presenting the results as fold uptake compared to the sham group showed the reverse trend. For a PNP of 500 kPa, the largest molecule of 70 kDa yielded a 21.5 fold higher uptake compared to the sham group, whereas for the 1.2 kDa molecule, a 2.8 fold uptake was observed. This is due to the fact that the uptake in the sham groups decreased significantly as the molecular weight of the delivered molecule increased. The fraction of fluorescent cells was used here as the metric to evaluate the delivery efficacy and compare between the different molecules delivery. Alternatively, the number of molecules that were taken up by the cells can be quantified for drug delivery and therapeutic outcome assessment.

The experiments were conducted in Eppendorf tubes due the physical dimensions of the transducer's focal spot. For the center frequency of 250 kHz, the full width at half maximum for the lateral and axial axes were 7 × 50 mm, respectively. Taking advantage of the Eppendorf and focal elongated shapes, enables to conduct the treatment in these tubes, while treating the entire volume simultaneously without the need to mechanically move the transducer, as required in case of using adherent cells in plates. In vivo, the TMB can either be intravenously injected into the blood vessels, and interact with the endothelial cells, or can be locally injected directly into the tumor, and bind to cancer cells. The scenario that is mimicked in vitro resembles the intratumoral injection (Bismuth et al., 2021). Under this condition, the entire tumor volume can be treated simultaneously using a single low frequency focused US application. The cell culturing is performed in plates, and following the US treatment, the cells are transferred back to 24-well plates. In addition, the same procedures that the treated groups undergo, are conducted also in the control groups.

The PNPs ranged from 0 to 800 kPa. The upper limit was chosen to remain below the FDA mechanical index limit of 1.9 (Chowdhury et al., 2020; Bader and Holland, 2012). For all molecule sizes, at low PNPs, the fraction of fluorescent cells increased as a function of the PNP. However, beyond a PNP of 500 kPa, increasing the PNP to 800 kPa did not increase the fluorescent cells percentage significantly. This suggests that 500 kPa is the optimal PNP for sonoporation at a center frequency of 250 kHz. The expected expansion ratio for a PNP of 500 kPa is 33, and for 800 kPa is 50. These high expansions are both likely to create large pores in the cell membrane, thus increasing the PNP to 800 kPa, which does not affect delivery efficacy, but reduces cell viability.

Viability experiments were performed at three time points that match the fluorescence experiments time points, due to technical differences between the dyes and procedures. The smallest molecule (7-AAD, 1.2 kDa) is a fluorescent dye that undergoes a spectral shift upon association with DNA. Therefore, following sonoporation, the fluorescent signal arises only from the stained cells and does not exist in the background suspension. Consequently, fluorescence microscopy can be used immediately after the treatment to visualize and quantify the percentage of fluorescent cells. Unlike 7-AAD, FITC-dextran fluoresces on its own, and high background signal existed in the background suspension immediately after treatment. In order to remove background suspension, after the US treatment, the cells suspension was cultured for additional 24 h in plates. During this time, the cells adhered to the plate, and the media was washed and replaced to remove all fluorescent background signal. Therefore, 4, 20 and 70 kDa FITC-dextrans fluorescence and viability tests were performed 24 h post treatment. An additional viability test was performed 72 h post treatment to evaluate cells recovery as a function of time. Immediately after treatment, cell viability was ∼40% for all of the treatment groups. However, it dropped to below 20% after 24 h. This suggests that cell death post- sonoporation requires time. Importantly, cells recovered their viability 72 h post-treatment, where viability increased to 52% for the group treated with 500 kPa US and TMB. For cancer cell sonoporation, low viability is an advantage to reduce tumor burden. Therefore, a similar optimization needs to be achieved for sonoporation of other types of cells.

In vitro studies are considered a prerequisite prior to in vivo studies. The optimization results obtained here will be used in future in vivo studies, to assess delivery efficacy in a tumor model in mice. In vivo, additional factors could affect sonoporation efficacy such as the surrounding media's viscoelasticity (Yang et al., 2020), cell shape and connections (Park et al., 2011), and also individual patients' variations in age, body mass index and many other factors. However, previous studies with 250 kHz US insonation have shown good agreement between in vitro and in vivo results (Ilovitsh et al., 2020; Bismuth et al., 2021). This model can be used to not only identify the relationship between the delivered molecule size and the insonation parameters, but also to adjust the sonoporation parameters to different tumors in different subjects. In addition, with the vast amount of multidrug treatments that are increasingly used, this platform can facilitate the comparison between different molecules delivery ranging from smaller chemotherapeutic drugs but also for larger complex drug carriers.

## 5 Conclusion

This study optimized low frequency sonoporation efficacy by identifying the relationship between the delivered molecule size and the insonation parameters using the same acoustical setup. The development of low frequency MB-mediated ultrasound can be used as a highly effective drug delivery platform of large molecules with high spatiotemporal precision. The results confirmed that the optimal PNP for molecule delivery at a center frequency of 250 kHz is 500 kPa. The highest fraction of fluorescent cells was obtained for the smallest molecule, and dropped as the molecule size increased. However, since the baseline in the sham group was the lowest, delivery uptake presented in folds compared to sham was the highest for the largest molecule. Overall, the use of low frequency and low MI enables the efficient delivery of different sized molecules, while reducing cancer cell viability. This approach can be used in the future as a combined method to maximize the therapeutic effects of cancer treatment.

## Notes

The authors have no competing interests to declare.

## Declaration of Competing Interest

None.

## Data Availability

Data will be made available on request.

## Appendix A Supplementary data

Supplementary material Image 1
