# Supplementary material for: Efficacy optimization of low frequency microbubble-mediated sonoporation as a drug delivery platform to cancer cells
Source: Int J Pharm X. 2022 Sep 22;4:100132. doi: 10.1016/j.ijpx.2022.100132 (PMC9520274; doi:10.1016/j.ijpx.2022.100132)
Supplement: Supplementary file 1 — Supplementary material [file mmc1.pdf]

Supplementary Information

Efficacy optimization of low frequency microbubble-mediated sonoporation as a drug delivery platform to cancer cells

Michal Eck [a], Ramona Aronovich [a], Tali Ilovitsh [a,*]

[a] Department of Biomedical Engineering, Tel Aviv University, Tel Aviv 6997801, Israel.

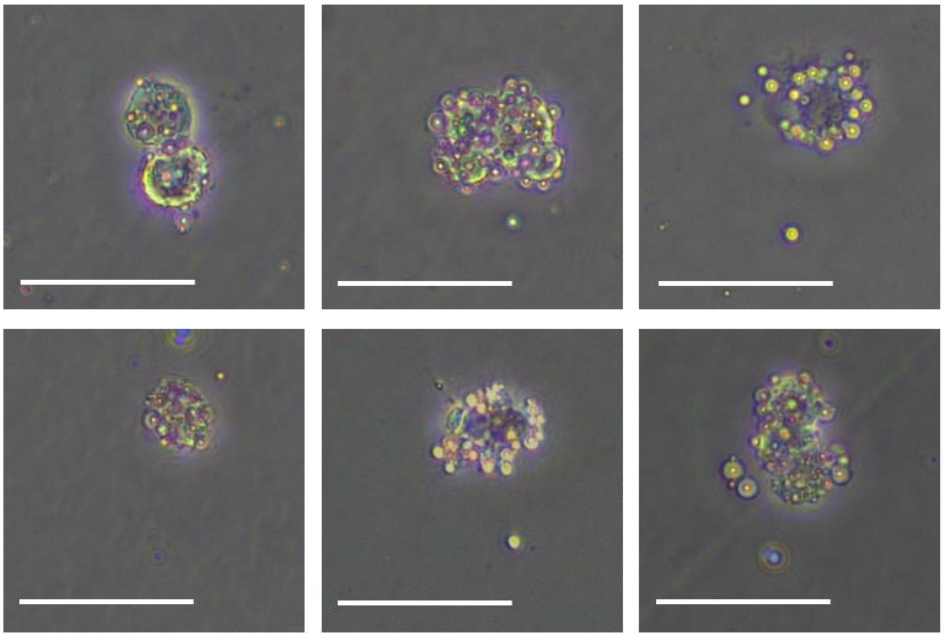

Fig. S1. Microscope images showing 4T1 cells with targeted microbubbles attached to the cells, at 20X magnification. Scale bar is common to all images and is 50 µm.
